# Safety, efficacy, and acceptability of ADV7103 during 24 months of treatment: an open-label study in pediatric and adult patients with distal renal tubular acidosis

**DOI:** 10.1007/s00467-020-04873-0

**Published:** 2021-02-26

**Authors:** Aurélia Bertholet-Thomas, Catherine Guittet, Maria A. Manso-Silván, Sophie Joukoff, Victor Navas-Serrano, Véronique Baudouin, Mathilde Cailliez, Massimo Di Maio, Olivia Gillion-Boyer, Emilija Golubovic, Jérôme Harambat, Bertrand Knebelmann, François Nobili, Robert Novo, Ludmila Podracka, Gwenaëlle Roussey-Kesler, Luc-André Granier

**Affiliations:** 1 Centre de Référence des Maladies Rénales Rares–Néphrogones–Hôpital Femme Mère Enfant, Hospices Civils de Lyon–Filière ORKiD, Bron, France; 2 Advicenne, Nîmes, France; 3 Service de Néphrologie Pédiatrique, Hôpital Robert Debré, Paris, France; 4 Service de Pédiatrie Multidisciplinaire, Hôpital de la Timone, AP-HM, Marseille, France; 5 Service de Réanimation Néonatale et Néonatologie, CHU de Nîmes, Nîmes, France; 6 Service de Néphrologie Pédiatrique, Centre de Référence des Maladies Rénales Héréditaires de l’Enfant et de l’Adulte (MARHEA), Institut Imagine, Hôpital Necker-Enfants Malades, Université de Paris, Paris, France; 7 Klinika za dečije interne bolesti–Odeljenje za nefrologiju, Klinički Centar Niš, Niš, Serbia; 8 Service de Pédiatrie, Centre de Référence Maladies Rénales Rares du Sud-ouest (SoRare), CHU de Bordeaux, Hôpital Pellegrin-Enfants, Bordeaux, France; 9 Service de Néphrologie adultes Hôpital Necker, Paris, France; 10 Service de Pédiatrie 2, Hôpital Jean Minjoz, CHU de Besançon, Besançon, France; 11 Service de Néphrologie Pédiatrique, Hôpital Jeanne de Flandre, CHRU de Lille, Lille, France; 12 Department of Pediatrics, National Institute of Children’s Health, Bratislava, Slovakia; 13 Unité de Néphrologie et Hémodialyse Pédiatrique, Clinique Médicale Pédiatrique Hôpital Mère-Enfant, CHU de Nantes, Nantes, France

**Keywords:** dRTA, Safety, Plasma bicarbonate, Acceptability, Adherence to treatment

## Abstract

**Background:**

A new prolonged-release formulation of potassium citrate and potassium bicarbonate, ADV7103, has been shown to improve metabolic control, palatability, and gastrointestinal safety in patients with distal renal tubular acidosis (dRTA) when compared to standard of care (SoC) treatments. The present work evaluates safety and efficacy of ADV7103 during 24 months.

**Methods:**

Thirty pediatric and adult patients were included in an open-label extension study after a phase II/III trial. Safety and tolerability were assessed. Plasma bicarbonate and potassium levels, as well as urine parameters, were evaluated over time. Acceptability, adherence, and quality of life were also assessed. The evolution of clinical consequences of dRTA in the cohort was explored.

**Results:**

There were 104 adverse events (AEs) reported, but only 9 gastrointestinal events observed in five patients (17%) were considered to be related to ADV7103 treatment. There were no AEs leading to treatment discontinuation. Plasma bicarbonate and potassium levels were in the normal ranges at the different visits, respectively, in 69–86% and 83–93% of patients. Overall adherence rates were ≥ 75% throughout the whole study in 79% patients. An average improvement of quality of life of 89% was reported at 24 months of study.

**Conclusions:**

Common AEs concerned metabolism and gastrointestinal disorders; the former being related to the disease. Less than half of the gastrointestinal AEs were related to ADV7103 treatment and they were mostly mild in severity. Metabolic parameters were maintained in the normal ranges in most patients. Patient satisfaction was high and adherence to treatment was good and remained stable.

**Trial registration number:**

Registered as EudraCT 2013-003828-36 on the 3rd of September 2013.

**Graphical Abstract:**

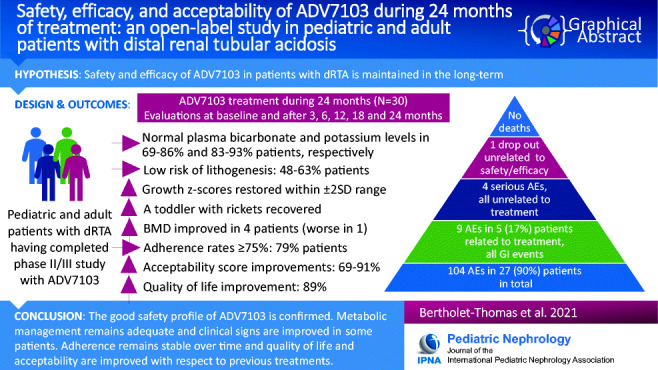

**Supplementary Information:**

The online version contains supplementary material available at 10.1007/s00467-020-04873-0.

## Introduction

Treatment of distal renal tubular acidosis (dRTA), a rare disorder characterized by hyperchloremic metabolic acidosis, frequently combined with hypokalemia, aims at correcting biochemical abnormalities in plasma, and subsequently maintaining biochemical parameters within acceptable limits in order to avoid their clinical consequences [1–7]. Current treatment of dRTA consists of alkalizing salts that are not licensed for dRTA (with no clinical development in the disease). Available standard of care (SoC) treatments present inconvenient dosing schemes, bad taste, frequent gastrointestinal side effects, low treatment adherence, and sub-optimal efficacy [5]. In a recent retrospective study including 31 Turkish patients, all treated with potassium citrate and/or sodium bicarbonate, only 13% of patients presented adequate metabolic control in more than 75% of the visits [8]. Additionally, available formulations are not always adapted to pediatric use.

A new oral granule formulation (ADV7103), which combines the advantages of potassium citrate and potassium bicarbonate, provides an effective prolonged release of alkalizing salts affording a 12-h effect with adequate tolerability [9].

A multicenter, open-label, non-inferiority phase II/III trial, comparing ADV7103 with SoC treatments in patients with a confirmed diagnosis of dRTA, has been recently published [10]. Non-inferiority of ADV7103 versus SoC regarding plasma bicarbonate levels, a biological marker of dRTA, was shown and its statistical superiority was demonstrated. The number of responders with normal mean plasma bicarbonate values was significantly increased when switching to ADV7103, reaching a response rate of 90%, while it was of 43% previously with SoC. Plasma potassium levels were in the normal range in 83% of patients and significant improvement of other parameters, such as urine calcium/citrate ratio, palatability, and gastrointestinal tolerability, was also reported [10].

The aim of the present study was to assess safety, tolerability, and efficacy of ADV7103, as well as to evaluate adherence to treatment in adult and pediatric patients with dRTA after 24 months of treatment. Other clinical outcomes were investigated, including changes in nephrocalcinosis, nephrolithiasis, estimated glomerular filtration rate (eGFR), bone remodeling, rickets, osteomalacia, and growth in children, along with treatment acceptability and quality of life.

## Methods

### Study design and patient population

**Fig. 1:**
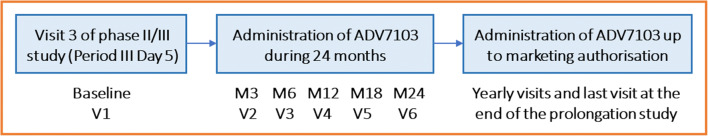
Study design

At the end of the previous phase II/III study [10], all patients with dRTA who had participated and completed the study were invited to participate in a multicenter, single-arm, open-label, follow-up study (EudraCT 2013-003828-36). Adults (18 to 55 years old), adolescents (12 to 17 years old), children (4 to 11 years old), and infants and toddlers (6 months to 3 years old) were enrolled to continue their treatment with ADV7103 for at least 24 months at the optimal dose previously determined and further adapted if required. ADV7103 consists of prolonged-release granules, presented in 8 mEq and 24 mEq sachets containing roughly one part of potassium citrate and two parts of potassium bicarbonate [9]. The total daily dose was divided in two intakes, one in the morning and another in the evening, including one or several sachets. The granules in each sachet were swallowed with a glass of water or taken in several portions with small amounts of soft food (e.g., mixed fruits, yoghurt), without chewing or crushing. Exclusion criteria were unusual additional proximal tubular signs, hyperkalemia (plasma potassium > 5.0 mmol/L), moderate or severe kidney impairment (eGFR < 45 mL/min/1.73 m^2^), or any other condition that could be negatively affected by the study medication or that could affect the study medication. Patients receiving potassium-sparing diuretics, angiotensin-converting enzyme inhibitors, angiotensin II receptor antagonists, or tacrolimus were also ruled out. Study visits were scheduled at baseline (last visit of phase II/III trial) and after 3, 6, 12, 18, and 24 months of treatment (Fig. 1). Thereafter, one visit per year was scheduled for patients continuing beyond the 24 months (not reported in the present publication).

### Safety and tolerability

The primary objective of the study was to evaluate the long-term safety of ADV7103 as measured by the occurrence of adverse events (AEs). Numbers and percentages of patients presenting AEs were tabulated by body system and preferred term, according to the Medical Dictionary for Regulatory Activities (MedDRA, Version 18.0). Severity of the adverse events was determined. Abnormal values on vital signs, ECG parameters, physical examination, and laboratory data were also recorded during the course of the study.

### Blood parameters

Plasma bicarbonate and potassium levels were assessed at each study visit. They were measured in blood samples generally drawn before first morning dose of treatment. Metabolic acidosis was considered when plasma bicarbonate level was below the normal range defined by the local laboratory (lower boundary was generally 20 to 22 mmol/L, depending on the age of the patients) and hypokalemia was considered when plasma potassium level was below the normal range defined by the local laboratory (generally 3.5 mmol/L, but could be as low as 3.1 mmol/L for pediatric patients). Details about methods and normal ranges are provided as supplementary information.

### Urine parameters and renal exploratory evaluations

Urine excretion parameters, crystalluria, and eGFR were measured at each study visit. Hypercalciuria was considered when urine calcium/creatinine excretion ratio (UCa/UCr, mmol/mmol) was above the corresponding age-specific normal range [11, 12] and hypocitraturia was considered when urine citrate/creatinine excretion ratio (UCi/UCr, mmol/mmol) was below the corresponding age-specific normal range [13] (see Supplementary Information). Increased risk of lithogenesis was considered when the urine calcium/citrate excretion ratio (UCa/UCi, mmol/mmol) was above the threshold for risk of lithogenesis of 3 mmol/mmol [14]. Positive crystalluria was defined as the presence of at least one crystal species. The presence of nephrocalcinosis and nephrolithiasis was evaluated at baseline and after 24 months of study.

### Adherence to treatment

Adherence was estimated based on accountability of study drug retrieved. The proportion of treatment that had been taken versus what should have been taken was calculated.

Adherence was indicated at each study visit up to 24 months as one of four possible adherence categories: excellent (> 90%), good (75–90%), average (50–74%), and poor (< 50%). In some particular cases, when drug accountability was not possible (e.g., the patient did not bring the empty treatment boxes), the investigators could class the patient in one of the four categories on the basis of laboratory results and interview of the patient and their parents.

### Growth parameters, bone remodeling, and other exploratory evaluations

Height, weight, and body mass index (BMI) were evaluated at each study visit and the corresponding *z*-scores were determined [15] according to WHO standards [16, 17], considering age and sex. Values were considered normal when they were within the ± 2SD range.

Bone mineral density (BMD, g/cm^2^) was measured separately for three skeletal regions (spine, hip, and whole body) at baseline and after 24 months of treatment and the corresponding *z*-scores were provided. The *z*-score was considered below the expected range for a value ≤ − 2.0 according to recommendations of the International Society for Clinical Densitometry (ISCD) [18]. An overall clinical interpretation was provided.

Specific blood markers were analyzed at each study visit, by a central laboratory (bone alkaline phosphatases, 1,25-dihydroxy-vitamin D, and parathyroid hormone) or by local laboratories (phosphate, calcium, and 25-hydroxy-vitamin D). Details about the methods used and normal ranges considered by each local laboratory are provided as supplementary information.

The presence of rickets and osteomalacia was evaluated at baseline and after 24 months of study, based on clinical signs, such as diffuse bone and joint pain, myalgia, muscle weakness, abnormal posture, and abnormal stature, and biochemical signs, including abnormal levels of alkaline phosphatases, calcium, and phosphate.

### Quality of life and acceptability

Quality of life (QoL) and treatment acceptability were explored through questions about the perception of the improvement of different parameters when compared to SoC treatments taken previously. They were assessed by the patients (or their parents) with 100-mm visual analogue scales (VAS, where 0 meant “No improvement at all” and 100 meant “Extremely high improvement”). Improvement of QoL was evaluated for patients after 6 and 24 months and for their parents after 24 months of treatment. In order to evaluate acceptability, improvement of the number of daily intakes, appropriateness of the formulation, taste, efficacy, and gastrointestinal tolerability, were assessed at the 24-month visit.

### Statistical methods

The primary endpoint was the number/proportion of patients presenting AEs during the course of the study, including the incidence and severity of these events. The safety analysis (SA) set included all patients who received at least one dose of study drug.

The efficacy analysis (EA) set included all patients who received at least one dose of study drug and who had at least one efficacy assessment. However, subgroup analyses were performed with patients for whom it could be ascertained that blood samples were drawn before first morning dose. For plasma potassium determinations, patients with hemolyzed blood samples were tagged in order to perform subgroup analyses excluding them. The number/proportion of patients presenting normal values for plasma or urine parameters was calculated at each time point.

The QoL analysis (QA) set included all patients who received at least one dose of study drug and who had at least one QoL assessment.

Safety, efficacy, adherence, and QoL data and their change from baseline (when appropriate) were summarized by age group, overall, and over time using descriptive statistics.

Statistical analyses were generated using SAS software, Version 9.4, of the SAS System for Windows (SAS is a registered trademark of SAS Institute Inc., Cary, NC, USA).

## Results

A total of 30 patients (93.8% of the patients who completed the pivotal study), all with primary dRTA, were enrolled (Table 1) in 12 different centers in France, Serbia, and Slovakia. One patient dropped out after 12 months of treatment for personal reasons. Among the patients enrolled, 66.7% presented hearing impairment. One toddler had hypokalemia, two patients (one child and one adult) had osteopenia, and one child had polyuria.

**Table 1:** Patient disposition and summary of demographic data by age group and overall

	Adults	Adolescents	Children	Infants and toddlers	Overall
*N* enrolled	6	8	13	3	30
*N* (%) females	4 (67%)	6 (75%)	7 (54%)	0 (0%)	17 (57%)
*N* (%) males	2 (33%)	2 (25%)	6 (46%)	3 (100%)	13 (43%)
Age (Y)					
Median (range)	19.3 (18.8, 21.6)	14.0 (12.7, 17.3)	6.5 (4.6, 11.6)	3.4 (2.1, 3.8)	10.3 (2.1, 21.6)
Weight (kg)					
Median (range)	59.3 (50.2, 87.0)	44.3 (41.0, 57.7)	21.5 (12.4, 50.0)	15.3 (12.0, 20.2)	41.1 (12.0, 8 7.0)
Weight (*z*-score)	NA	NA			
Median (range)			− 0.2^a^ (− 2.5, 2.6)	0.1 (− 0.3, 1.8)	− 0.1^a^ (− 2.5, 2.6)
Height (cm)					
Median (range)	160 (149, 169)	161 (146, 170)	116 (92, 146)	101 (86, 102)	141 (86, 170)
Height (*z*-score)					
Median (range)	− 1.4^b^ (− 1.8, − 1.0)	− 0.4 (− 1.2, 1.5)	− 0.6 (− 3.3, 0.8)	0.0 (− 0.9, 0.5)	− 0.5^b^ (− 3.3, 1.5)
BMI (kg/m^2^)					
Median (range)	23.9 (19.9, 30.5)	16.9 (15.5, 24.2)	16.0 (12.8, 23.5)	16.4 (15.0, 19.4)	17.3 (12.8, 30.5)
BMI (*z*-score)					
Median (range)	0.7^b^ (− 0.8, 2.2)	− 1.1 (− 2.0, 1.3)	0.1 (− 2.3, 3.3)	0.4 (− 0.4, 2.8)	− 0.1^b^ (− 2.3, 3.3)
*N* completed	5	8	13	3	29

*N* number of patients, *Y* years

^a^Weight *z*-scores available for patients ≤ 10Y

^b^Height and BMI *z*-scores available for patients ≤ 19Y

### Adverse events and safety and tolerability observations

A total of 27 patients (90%) experienced the 104 AEs observed during the course of the study: 20 AEs in four adult patients (67%), 37 AEs in eight adolescents (100%), 37 AEs in twelve children (92%), and 10 AEs in three toddlers (100%), most of them (83 AEs in 23 patients) of mild intensity. As shown in Table 2, there were 21 metabolism and nutrition AEs in 14 patients (47%), 20 gastrointestinal AEs in 12 patients (40%), and 29 infections and infestations in 10 patients (33%). A low frequency of AEs was reported (< 10 AEs) in the remaining body systems.

**Table 2:** Adverse events by body system

Body system	Number of adverse events	Number of patients with at least one adverse event	Percentage of patients with at least one adverse event
Blood and lymphatic system disorders	1	1	3.3
Endocrine disorders	1	1	3.3
Gastrointestinal disorders	20	12	40.0
General disorders and administration site conditions	1	1	3.3
Immune system disorders	1	1	3.3
Infections and infestations	29	10	33.3
Injury, poisoning and procedural complications	2	2	6.7
Investigations	2	2	6.7
Metabolism and nutrition disorders	21	14	46.7
Musculoskeletal and connective tissue disorders	6	6	20.0
Nervous system disorders	3	3	10.0
Kidney and urinary disorders	8	5	16.7
Reproductive system and breast disorders	2	2	6.7
Skin and subcutaneous tissue disorders	5	5	16.7
Surgical and medical procedures	2	2	6.7
TOTAL	104	27	90.0

There were no AEs leading to treatment discontinuation and no deaths reported during the study. Four serious AEs were reported in four patients (13.3%), all considered unrelated to treatment: renal colic, acute gastritis, wisdom teeth removal, and rotavirus gastroenteritis, which resolved within the following days without modification of the dose.

Only nine AEs noted in five patients (16.7%), all gastrointestinal AEs, were considered related to the treatment: three events of diarrhea in one patient, two events of abdominal pain in two children, and single events of dyspepsia, upper abdominal pain, gastrointestinal disorder, and gastrointestinal pain. They were of mild or moderate severity and resolved without discontinuation of the treatment. Only one of them (gastrointestinal disorder in an adolescent) required a dose adjustment, which was without consequence on plasma and urine metabolic parameters of the patient. Concomitant treatments were provided in certain cases, such as intestinal absorbent clay for diarrhea, gastrointestinal pain and gastrointestinal disorder, antacid for abdominal pain, and an antispastic for dyspepsia.

There were no clinically significant abnormalities in vital signs. No episodes of hyperkalemia were reported and there were no ECG abnormalities, including those classically associated with hyperkalemia. Physical examination findings were normal except for a non-clinically significant weight gain at month 18 in a child and a molluscum contagiosum in another child, both declared as unrelated to treatment.

Among the abnormalities that were considered to be clinically significant after clinical laboratory evaluations, most were cases of low blood levels of 25-hydroxyvitamin D (three adolescents, two children, and one infant). At baseline, the overall mean ± SD blood 25-hydroxy-vitamin D level was 51.5 ± 33.7 nmol/L but was as low as 20 nmol/L in one of the patients. At the 24-month visit, mean ± SD was 58.9 ± 20.8 nmol/L and the lowest value observed was 27.5 nmol/L. Sixty percent of the patients received vitamin D supplementation at some time point during the study. One child had clinically significant high erythrocyte and leucocyte levels on microscopic analysis of urine.

### Evolution of the dose

The overall mean ± SD dose at baseline was 107.7 ± 57.4 mEq/day and increased up to 117.0 ± 67.6 mEq/day after 24 months of treatment. The overall mean ± SD dose per body weight was 3.4 ± 1.7 mEq/kg/day at baseline and 3.2 ± 1.6 mEq/kg/day at month 24. This represented an average dose of 2.3 ± 1.3 mEq/kg/day in the adult group, 2.6 ± 1.7 mEq/kg/day in the adolescent group, 3.4 ± 1.3 mEq/kg/day in the child group, and 4.8 ± 2.0 mEq/kg/day in the infant/toddler group, respectively, by month 24.

### Metabolic parameters and renal consequences of dRTA

**Fig. 2:**
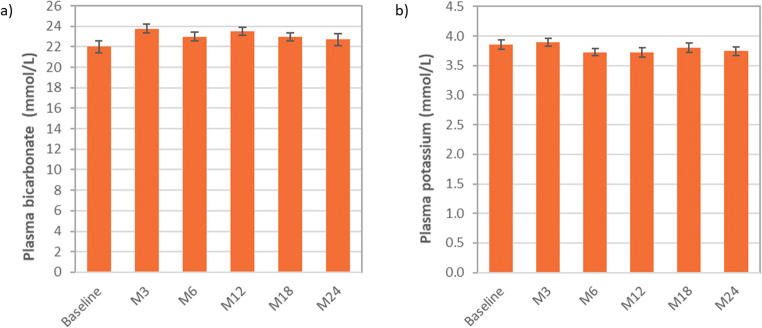
Mean (SEM) overall values of **a** plasma bicarbonate and **b** plasma potassium with ADV7103 at baseline and after 3, 6, 12, 18, and 24 months of treatment

The evolution of overall mean plasma bicarbonate and plasma potassium levels is shown in Fig. 2. Similar results were obtained in the subgroup analyses. At 24 months, overall mean ± SD plasma bicarbonate levels were 22.7 ± 3.1 mmol/L considering all available data (*N* = 29) and 22.8 ± 2.9 mmol/L when considering only patients for whom blood samples were drawn before first morning dose (*N* = 24). Plasma potassium levels were 3.7 ± 0.4 mmol/L considering all available data (*N* = 29) and 3.8 ± 0.3 mmol/L considering only patients for whom blood samples were drawn before first morning dose and with non-hemolyzed samples (*N* = 23). Plasma bicarbonate was in the normal range in 63% of patients at baseline and in 69 to 86% of patients throughout the 24 months of treatment. Only one adult patient was considered to have clinically significant metabolic acidosis at month 18 and month 24. Plasma potassium was in the normal range in 90% of patients at baseline and in 83 to 93% of patients at following visits. One child and an adolescent at month 12 and an adult at month 24 were considered to present clinically significant hypokalemia.

**Fig. 3:**
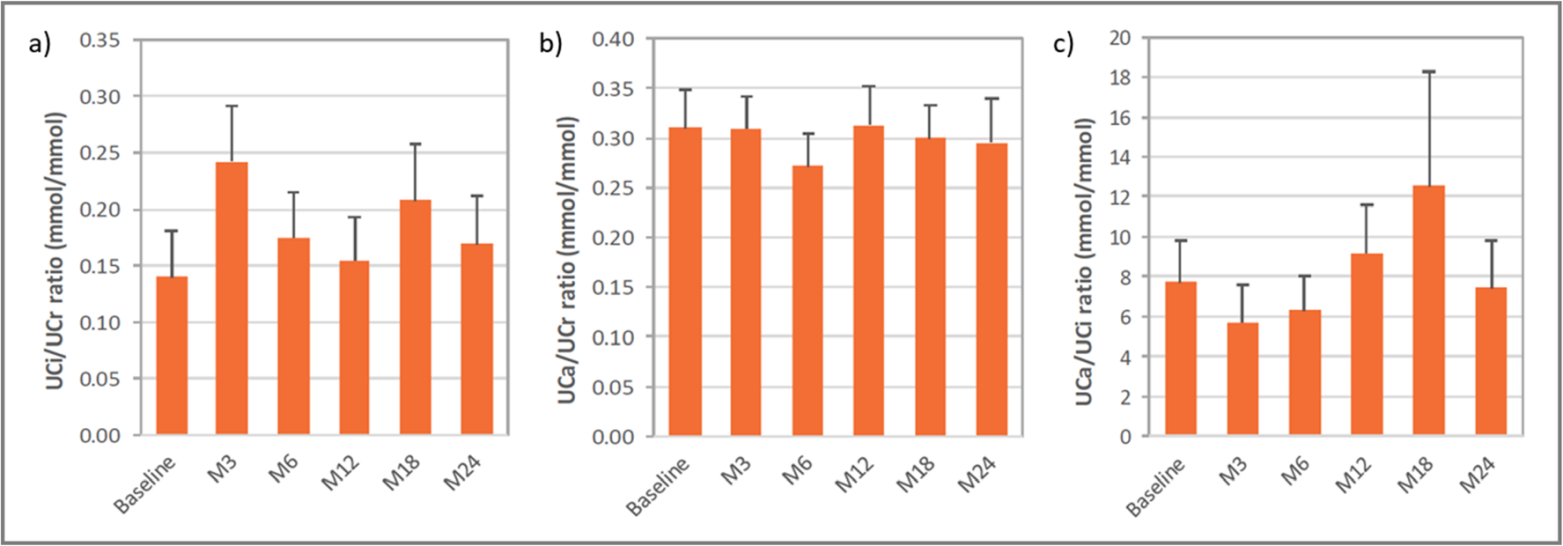
Mean (SEM) overall values of **a** urine citrate/creatinine ratio, **b** urine calcium/creatinine ratio, and **c** urine calcium/citrate ratio with ADV7103 at baseline and after 3, 6, 12, 18, and 24 months of treatment

The evolution of overall mean urine parameters is shown in Fig. 3. The overall mean ± SD UCi/UCr ratio was 0.2 ± 0.2 mmol/mmol at 24 months (change from baseline of − 0.01 ± 0.18 mmol/mmol), with values generally above 0.3 mmol/mmol in infants/toddlers and below 0.1 mmol/mmol in adults. At the different visits, 9 to 17 patients presented hypocitraturia. Overall mean ± SD UCa/UCr ratio was 0.3 ± 0.2 mmol/mmol at 24 months (change from baseline of − 0.06 ± 0.14 mmol/mmol). With the exception of one or two cases of hypercalciuria (affecting 5 different patients in all) at each time point, patients presented values of UCa/UCr within the normal range for their age throughout the 24 months. The overall mean ± SD UCa/UCi ratio was 7.4 ± 11.5 mmol/mmol at 24 months (change from baseline of 0.6 ± 8.9 mmol/mmol). The percentage of patients with UCa/UCi ratio below the threshold for risk of lithogenesis was 45% at baseline and ranged from 48 to 63% at all other visits.

At baseline, 9 patients had a positive crystalluria result. Amorphous carbonated calcium phosphate (ACCP) crystals were observed in 8 patients and an unknown type of crystal was reported in one infant. After 24 months of treatment, ACCP crystals were reported in 7 cases out of the 8 observed at baseline; ammonium hydrogen urate crystals were reported in three cases; brushite, struvite, and rare calcium oxalate crystals were reported each in one case.

Only six patients (20.7%) presented nephrolithiasis at baseline, from which one child recovered after 24 months of treatment. A total of 25 patients (86.2%), 5 adults, 6 adolescents, 13 children, and 1 toddler at baseline, and 28 patients (96.6%), 5 adults, 7 adolescents, 13 children, and 3 toddlers at month 24, presented nephrocalcinosis.

Mean ± SD values of eGFR were 116.2 ± 19.8, 106.5 ± 12.8, 121.2 ± 19.2, and 128.0 ± 11.4 mL/min/1.73 m^2^ in adults, adolescents, children, and toddlers, respectively, after 24 months of treatment. Considering paired patient data, the overall mean ± SD change from baseline in eGFR was of − 5.6 ± 24.3 mL/min/1.73 m^2^, with mildly decreased kidney function in a single patient at the last study visit (there was already one at baseline).

### Treatment adherence, improvement of quality of life, and acceptability

Treatment adherence values over time are shown in Table 3. Adherence rates were ≥ 75% in 79.3% of patients remaining in the study at month 24 (100% of adults, 62.5% of adolescents, 84.6% of children, and 2 out of the three toddlers, respectively).

**Table 3:** Number of patients (%) for each adherence category over time

Adherence category	Baseline–3 M (*N* = 30)	3–6 M (*N* = 29)	6–12 M (*N* = 30)	12–18 M (*N* = 29)	18–24 M (*N* = 29)
Excellent	22 (73.3)	19 (65.5)	22 (73.3)	19 (65.5)	18 (62.1)
Good	6 (20)	7 (24.1)	3 (10)	4 (13.8)	5 (17.2)
Average	1 (3.3)	2 (6.9)	4 (13.3)	5 (17.2)	6 (20.7)
Poor	1 (3.3)	1 (3.4)	1 (3.3)	1 (3.4)	0

*N*, number of patients; *M*, months

**Fig. 4:**
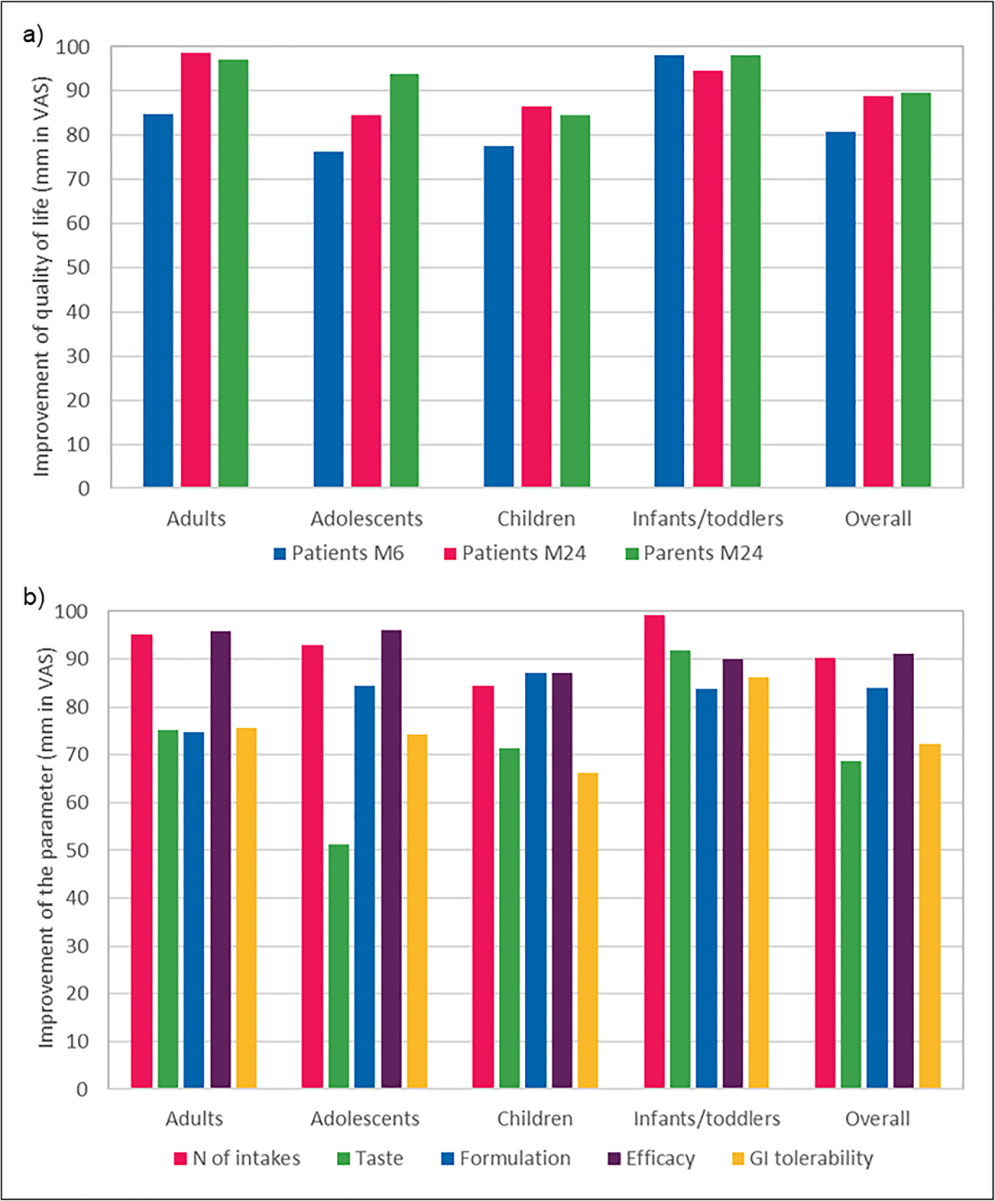
Improvement of **a** quality of life at 6 and 24 months, and **b** number of intakes, taste, formulation, efficacy, and gastrointestinal tolerability at 24 months after change of alkalizing treatment from SoC to ADV7103 in the different age groups and overall

As shown in Fig. 4a, the change of alkalizing treatment from SoC to ADV7103 led to an overall average improvement in the QoL of the patients of 80.7% at month 6 and of 88.9% at month 24, and of their parents of 89.6% at month 24.

Regarding acceptability, overall scores indicated improvements of 90.2% for the number of daily intakes, 83.9% for appropriateness of the formulation, 68.6% for taste, 91.2% for efficacy, and 72.2% for gastrointestinal tolerability (see Fig. 4b).

### Growth and bone mineral density

Most patients presented *z*-scores within the ± 2SD range for weight, height, and BMI. Among pediatric patients, one child was below the ± 2SD range for height at baseline (*z*-score < − 3) but was within range from M12, with a total increase of 1.8 units after 24 months of treatment. Two children were below the ± 2SD range for weight at baseline but one of them was within the ± 2SD range from M6 and the other from M18. Median height and weight *z*-scores increased in all pediatric age groups, with an overall median change (range) of 0.2 (− 0.6, 1.8) for height (*n* = 23) and 0.3 (− 0.4, 0.8) for weight (*n* = 11).

There was one case of rickets in a toddler at baseline, which had recovered at the 24-month visit. No cases of osteomalacia were observed during the whole study.

Overall clinical interpretation of exploratory bone mineral density results indicated that 15 (54%) patients presented abnormal bone status at baseline. Four of them (two adults, one adolescent, and one toddler) benefited from a modification of their bone status from abnormal to normal after 24 months of treatment. One adolescent had normal status at baseline and became abnormal at the end of the evaluation period. Median change from baseline values in spine *z*-scores was positive in all age groups, with an overall median change (range) of 0.3 (− 0.9, 1.3).

## Discussion

ADV7103 is a prolonged-release oral formulation, which combines the advantages of potassium citrate and potassium bicarbonate. Its efficacy in the management of plasma bicarbonate levels and its superiority when compared to current SoC treatments have recently been shown in patients with dRTA [10].

Among the long-term effects of the disorder, failure to thrive, rickets, and stunted growth in children, and osteomalacia/osteopenia in young adults, due to release of both phosphate and calcium from bone in an attempt to compensate chronic metabolic acidosis [19, 20] are the major bone issues. These are a direct consequence of metabolic acidosis, as illustrated in a retrospective study including 95 patients with primary dRTA by the significant difference in blood pH values between patients with and without bone disease (7.32 ± 0.06 vs. 7.37 ± 0.06, *p* = 0.01) [21], and are corrected with adequate metabolic control [8].

Adequate growth parameters and BMD were observed during the study. Although most patients were within the ± 2SD range for height and weight, an overall positive evolution was shown, with a particularly marked effect on the patients who were below the range at baseline. Improvement in BMD in some patients was also observed.

Hypercalciuria is frequent, affecting 15% of patients, as reported in an international cohort including 340 patients with dRTA [5]. Supersaturation and precipitation of poorly soluble calcium salts, due to increased levels of urine calcium excretion together with low citraturia levels in dRTA patients, lead to progression of nephrocalcinosis and nephrolithiasis, which have been observed, respectively, in 88% and in 20–42% of patients (depending on the mutation) [5]. This may impact kidney function, leading to chronic kidney disease starting from adolescence [22], and potentially evolving to stage 5 chronic kidney disease/kidney failure. Approximately 30% of the patients with dRTA present eGFR values < 90 mL/min/1.73 m^2^, according to different retrospective cohort studies [5, 8, 22], but in adult patients, the proportion may reach 82% [5]. The decrease in kidney function in patients with dRTA has been estimated to be around 3.5 mL/min/1.73 m^2^ per year in a cohort of children and young adults [8] and 0.8 mL/min/1.73 m^2^ per year in a cohort of adult patients [5]. Higher kidney function is observed in patients with adequate metabolic control compared to those not adequately controlled [5].

In a retrospective study including 95 adult and pediatric patients with dRTA, significantly higher calcium urine levels were shown in patients with lithiasis when compared to those without stones [21]. In untreated patients, average UCa/UCr levels of 1.3 mmol/mmol, approximately 3 to 4 times those of normal controls, and average UCi/UCr levels below 0.06 mmol/mmol, less than 1/3 of those of normal controls, are observed [23]. With ADV7103, urine calcium excretion was controlled and mean UCa/UCr values were maintained at constant levels around 0.3 mmol/mmol. Citraturia was higher than that observed in untreated patients and mean UCi/UCr values around 0.2 mmol/mmol were observed, but hypocitraturia remained in half of the patients or more. Compared to current SoC treatments, ADV7103 has been shown to increase from 20 to 60% the percentage of patients with UCa/UCi excretion ratios below the threshold considered for an increased risk of lithogenesis [10] and this percentage was maintained around 50% throughout the present study. After 24 months of treatment, the mean UCa/UCi ratio was 7.4 mmol/mmol with ADV7103, while this ratio may reach average levels of 169 mmol/mmol in untreated patients [23].

The frequency of nephrocalcinosis and nephrolithiasis in the present study was in agreement with the literature [5]. One child recovered from nephrolithiasis but nephrocalcinosis was not shown to be improved during the study. Although its progression may be prevented, nephrocalcinosis has been reported to be maintained despite limitation of hypercalciuria [24]. However, more time under ADV7103 treatment would be required to draw further conclusions. Kidney function remained normal in the majority of the patients, with mean eGFR values > 100 mL/min/1.73m^2^ in all age groups during the 24 months of treatment with ADV7103.

Low plasma potassium levels (< 3.5 mmol/L) associated with dRTA due to renal potassium wasting [19, 25, 26] are correctly managed with ADV7103 by virtue of its composition in potassium salts [10]. Hypokalemia can cause fatigue, but more importantly cardiac arrhythmias, paralysis, and even death [27–29]. The results of the present study demonstrate normal bicarbonate and potassium levels are maintained during the 24 months of treatment with twice daily administration of ADV7103.

For adequate management, it is important to consider that dose adjustments are required in order to adapt the amounts of alkali. As expected, total doses (expressed in mEq/day) increased due to growth and weight gain, although alkali requirements (expressed in mEq/kg/day) are reduced with age in patients with dRTA due to the strongest physiological acid metabolism occuring in young children compared to adolescents and adults [21]. The presence of children with plasma bicarbonate values below the normal range at some time-points could be partially explained by insufficient dose increases and could indicate that the frequency of visits during childhood should be higher in order to best adapt dosing to the evolving needs of the patients. As suggested by the results of the phase II/III study, the doses prescribed with ADV7103 can be titrated to the optimal dosing, while with SoC treatment dosing seems to be limited by tolerability and acceptability [10]. Additionally, vitamin D supplementation was frequently required for prevention or treatment of hypovitaminosis.

Gastro-intestinal disorders, well-known adverse effect of alkalizing products, were among the most common AEs observed. Overall, the safety of ADV7103 is very good and the cases of gastrointestinal AEs were mostly mild in severity and were reported in few patients (16.7%). No new safety concerns were raised and the only drop out was without relation to the treatment.

Present results obtained with ADV7103 confirm the good acceptability of the product and the adequate adherence to treatment. The patients and their parents indicated high improvement of their QoL. Treatment with ADV7103 continues in the 29 patients who continued to the end of the 24 months originally planned.

One of the limitations of the study is that, considering the small number of patients due to the orphan nature of the disorder and the broad range of ages included, most exploratory evaluations only allowed reporting some interesting observations regarding improvement in individual patients. Additionally, patients were already treated before entering the study and so it was extremely difficult to observe a significant evolution confirming clinical improvement.

In conclusion, the safety observations over 24 months during the extension study in patients with dRTA are consistent with the known safety of ADV7103. The sustained control of metabolic acidosis, hypokalemia, and urine parameters, together with the good treatment acceptability and adherence, confirms the observations made during the previous short-term phase II/III trial, reinforcing the safety and efficacy profile of the new drug.

## Supplementary Information

ESM 1 (DOCX 72 kb).

ESM 1 (PPTX 57.5 kb).

## Data Availability

Data available under request
