# Supplementary material for: Safety, efficacy, and acceptability of ADV7103 during 24 months of treatment: an open-label study in pediatric and adult patients with distal renal tubular acidosis
Source: Pediatr Nephrol. 2021 Feb 26;36(7):1765–74. doi: 10.1007/s00467-020-04873-0 (PMC8172410; doi:10.1007/s00467-020-04873-0)
Supplement: Supplementary file 1 — (DOCX 72 kb). [file 467_2020_4873_MOESM1_ESM.docx]

**Supplementary meterial**

[Abbreviations 1](#_Toc44422525)

[1. LABORATORY METHODS AND NORMAL RANGES: PLASMA AND BLOOD 2](#_Toc44422526)

[Laboratories related to site 001 2](#_Toc44422527)

[Laboratories related to site 002 2](#_Toc44422528)

[Laboratories related to site 003 3](#_Toc44422529)

[Laboratories related to site 005 4](#_Toc44422530)

[Laboratories related to sites 007 and 009 4](#_Toc44422531)

[Laboratories related to site 008 5](#_Toc44422532)

[Laboratories related to site 012 5](#_Toc44422533)

[Laboratories related to site 013 6](#_Toc44422534)

[Laboratories related to site 015 6](#_Toc44422535)

[Laboratories related to sites 202 and 301 7](#_Toc44422536)

[Central laboratory 8](#_Toc44422537)

[2. NORMAL RANGES: URINE RATIOS AND RISK OF LITHOGENESIS 9](#_Toc44422538)

# Abbreviations

d: days

m: months

w: weeks

y: years

M: male

F: female

Only ranges pertinent to study patients have been defined

# 1. LABORATORY METHODS AND NORMAL RANGES: PLASMA AND BLOOD

## Laboratories related to site 001

| **Laboratory name/address** | **Parameters (units)** | **Normal ranges** | | **Analysis method** |
| --- | --- | --- | --- | --- |
| Biopole 21 Site du Parc de l’Europe  Dijon, FRANCE | Potassium (mmol/L) | Age: 3 y | 3.5 - 5.1 | Direct potentiometry |
|  | Bicarbonate (mmol/L) | Age: 3 y | 22 - 30 | Spectroreflectometry |
| Pôle de Biologie Hospice civils de Lyon (hospital Laboratory).  Lyon, FRANCE | Potassium (mmol/L) | Age:  28 d - 2 y  2 - 15 y  > 15 y | 4.1 - 5.3  3.4 - 4.7  3.5 - 4.8 | Indirect potentiometry  (Abbott Architect) |
|  | Bicarbonate (mmol/L) | Age:  28 d - 15 y  15 - 60 y  > 60 y | 20 - 28  22 - 29  23 - 31 | PEP carboxylase UV |
|  | Phosphate (mmol/L) | Age :  1 - 5 y  5 - 13 y  M: 13 - 16 y  F: 13 - 16 y  16 - 19 y  > 19 y | 1.39 - 2.2  1.32 - 1.91  1.03 - 1.78  1.13 - 2.0  0.94 - 1.62  0.74 - 1.52 | Photometry UV  (Abbott Architect) |
|  | Calcium (mmol/L) | Age:  10 d – 2 y  2 – 12 y  12 – 60 y  > 60 y | 2.25 - 2.75  2.20 - 2.70  2.10 - 2.55  2.20 - 2.50 | Photometry Arsenazo III (Abbott Architect) |
|  | 25-OH vit D (nmol/L) | 40 - 125 | | Chemiluminescence  immunoassays |

##

## Laboratories related to site 002

| **Laboratory name/address** | **Parameters (units)** | **Normal ranges** | | **Analysis method** |
| --- | --- | --- | --- | --- |
| Bioaxome  Chemin de Saint Paul  Manduel, FRANCE | Potassium (mmol/L) | 3.5 - 5.1 | | Potentiometry |
|  | Bicarbonate (mmol/L) | 22 - 29 | | Spectrophotometry |
| Pole Biologie CHU de Nîmes (hospital Laboratory).  Nîmes, FRANCE | Potassium (mmol/L) | Age:  1m - 3 y  3 -15 y  Adults | 4.1 - 5.3  3.4 - 4.7  3.5 - 5.1 | Potentiometry |
|  | Bicarbonate (mmol/L) | Adults | 22 - 29 | PEP carboxylase UV |
|  | Phosphate (mmol/L) | Age:  1 - 3 y  3 - 6 y  6 - 9 y  M : 9 - 12 y  F : 9 - 12 y  M : 12 - 15 y  F : 12– 15 y  15 - 18 y  > 18 y | 1 - 1.95  1.05 - 1.8  1.00 - 1.8  1.05 - 1.85  1.05 - 1.7  0.95 - 1.65  0.90 - 1.55  0.85 - 1.6  0.81 - 1.45 | Spectrophotometry |
|  | Calcium (mmol/L) | Age:  10d - 2y  2 - 12y  12 - 18y  18 - 60y | 2.25 - 2.75  2.20 - 2.70  2.1 - 2.55  2.15 - 2.50 | Spectrophotometry |
|  | 25-OH vit D (nmol/L) | 75 - 175 | | Immunoluminometry |

##

## Laboratories related to site 003

| **Laboratory name/address** | **Parameters (units)** | **Normal ranges** | | **Analysis method** |
| --- | --- | --- | --- | --- |
| Laboratoire de la Trillade (BIOTOP)  Avignon, FRANCE | Potassium (mmol/L) | 3.0 - 4.5 | | Indirect potentiometry |
|  | Bicarbonate (mmol/L) | 20 - 28 | | PEP carboxylase UV |
| Bioaxome  Laboratoire de la chartreuse  Place de la Croix  Villeneuve-lès-Avignon, FRANCE | Potassium (mmol/L) | 3.4 – 4.5 | | Indirect Potentiometry |
|  | Bicarbonate (mmol/L) | 20 - 28 | | PEP carboxylase UV |
| Laboratoire Rey Port de Bouc  Port de Bouc, FRANCE | Potassium (mmol/L) | Age: 2 - 7 y | 3.5 - 5.0 | K Ion selective electrode (Beckman Coulter) |
|  | Bicarbonate (mmol/L) | Age: 2 - 7 y | 21 - 31 | CO_2_ ion selective electrode (Beckman Coulter) |
| LaboSud Provence  Port-de-Bouc, FRANCE | Potassium (mmol/L) | 3.5 - 5.1 | | Ion selective electrode indirect |
|  | Bicarbonate (mmol/L) | 20 - 28 | | PEP Carboxylase UV |
| Biologie médicale de l’APHM (hospital Laboratory).  Marseille, FRANCE | Potassium (mmol/L) | Age:  < 1 y  Adults | 3.7 - 5.2  3.4 - 4.5 | Indirect potentiometry |
|  | Bicarbonate (mmol/L) | Age:  1 - 6 m  Adults | 20 - 29  22 - 29 | PEP carboxylase UV |
|  | Phosphate (mmol/L) | Age:  30 d - 10 y  10 - 15 y  Adult | 0.95 - 1.75  0.95 - 1.65  0.81 - 1.45 | Phosphomolybdate UV |
|  | Calcium (mmol/L) | Age :  10 d - 2y  2 - 12y  12 - 18y  18 - 60y  60 - 90y  > 90y | 2.25 - 2.75  2.20 - 2.70  2.10 - 2.55  2.15 - 2.5  2.20 - 2.55  2.05 - 2.40 | NM-BAPTA |
|  | 25-OH-Vit D  (nmol/L) | Age:  2y - 15y | 75 - 250 | Electrochemiluninescence |

##

## Laboratories related to site 005

| **Laboratory name/address** | **Parameters (units)** | **Normal ranges** | | **Analysis method** |
| --- | --- | --- | --- | --- |
| Laboratoire de Biologie médicale Hopital Robert Debré (hospital laboratory).  Paris, FRANCE | Potassium (mmol/L) | Age:  1 m - 2 y  2 -15 y  > 15 y | 3.7 - 5.4  3.1 - 4.7  3.5 - 4.5 | Direct potentiometry Siemens (Advia 1800) |
|  | Bicarbonate (mmol/L) | Age :  1m - 2 y  2 - 15 y  > 15 y | 18 - 27  20 - 28  22 - 29 | PEP carboxylase UV |
|  | Phosphate (mmol/L) | Age:  1m-2y  2-15y  >15y | 1.5-2.30  1.30-1.85  0.87-1.5 | Phosphomolybdate UV Siemens (Advia) |
|  | Calcium (mmol/L) | Age :  1m-15y  >15y | 2.20-2.70  2.25-2.60 | O-cresolphtaleine complex Roche (Advia 1800) |
|  | 25-OH vit D  (μg/L) | 20 - 60 | | Chemiluninescence immunoassays  YSIS |

##

## Laboratories related to sites 007 and 009

| **Laboratory name/address** | **Parameters (units)** | **Normal ranges** | | **Analysis method** |
| --- | --- | --- | --- | --- |
| Laboratoire Alesia  Paris, FRANCE | Potassium (mmol/L) | Age: Adults | 3.5 – 5.0 | Potentiometry |
|  | Bicarbonate (mmol/L) | Age: Adults | 22 - 29 | Colorimetric (Roche) |
| Laboratoire d’Ozoir  Ozoir La Ferrière, FRANCE | Potassium (mmol/L) | Age: Adults | 3.5 – 5.1 | Potentiometry |
|  | Bicarbonate (mmol/L) | Age: Adults | 22 - 29 | Chemiluminescence Architect (Abbot) |
| Pole Biologie Médicale Hôpital Necker-enfants maladies (hospital Laboratory).  Paris, FRANCE | Potassium (mmol/L) | Age :  1 m - 13 y  Adults | 3.1 - 4.7  3.5 - 4.5 | Indirect potentiometry |
|  | Bicarbonate (mmol/L) | Age :  1 m - 3 y  3 - 15 y  Adults | 19 - 24  20 - 28  22 - 29 | PEP carboxylase UV |
|  | Phosphate (mmol/L) | Age:  3m - 2y  2y - 15y  adult | 1.50 - 2.30  1.30 - 1.85  0.85 - 1.50 | Enzymatic |
|  | Calcium (mmol/L) | Age :  1m - 13y  adult | 2.20 - 2.70  2.25 - 2.60 | Colorimetry |
|  | 25-OH Vit D  (μg/L) | 30 - 60 | | IDS-Isys 25 OHD |

##

## Laboratories related to site 008

| **Laboratory name/address** | **Parameters (units)** | **Normal ranges** | | **Analysis method** |
| --- | --- | --- | --- | --- |
| Pôle Biologie du CHU de Bordeaux (hospital Laboratory).  Bordeaux, FRANCE | Potassium (mmol/L) | Age:  3m - 7 y  > 7 y | 3.5 - 5.3  3.5 – 5.0 | Indirect potentiometry |
|  | Bicarbonate (mmol/L) | 23 - 29 | | PEP carboxylase UV |
|  | Phosphate (mmol/L) | Age:  7 d – 2 y  2 y - 18y  18 y - 130 y | 1.2 - 2.2  1.0 - 1.8  0.8 - 1.4 | Spectrophotometry |
|  | Calcium (mmol/L) | > 10 d | 2.2 - 2.65 | Colorimetry |
|  | 25-OH vit D  (ng/mL) | 30 - 100 | | Chemiluminescence |

##

## Laboratories related to site 012

| **Laboratory name/address** | **Parameters (units)** | **Normal ranges** | | **Analysis method** |
| --- | --- | --- | --- | --- |
| Laboratoire du marché  Audincourt, FRANCE | Potassium (mmol/L) | Age: > 18 y | 3.5 - 5.5 | Indirect Potentiometry |
|  | Bicarbonate (mmol/L) | Age: > 18 y | 20 - 31 | PEP Carboxylase UV |
| Laboratoire de Terre Rouge  Besançon, FRANCE | Potassium (mmol/L) | Age: > 18 y | 3.5 – 5.1 | Indirect potentiometry |
|  | Bicarbonate (mmol/L) | Age: > 18 y | 21 - 32 | Enzymatic |
| Laboratoire de Biologie Médicale CHRU de Besançon (hospital Laboratory)  Besançon, FRANCE | Potassium (mmol/L) | 3.5 - 5.1 | | Indirect potentiometry |
|  | Bicarbonate (mmol/L) | 21 - 32 | | PEP carboxylase UV |
|  | Phosphate (mmol/L) | 0.96 - 1.59 | | Spectrophotometry |
|  | Calcium (mmol/L) | 2.12 - 2.52 | | Colorimetry Arsenazo III |
|  | 25-OH vit D (ng/mL) | 30 - 100 | | Chemiluminescence immunoassays YSIS |

##

## Laboratories related to site 013

| **Laboratory name/address** | **Parameters (units)** | **Normal ranges** | | **Analysis method** |
| --- | --- | --- | --- | --- |
| Centre de Biologie CHRU de Lille  (hospital Laboratory).  Lille, FRANCE | Potassium (mmol/L) | Age:  1 - 18 y  > 18 y | 3.1 - 5.1  3.5 - 5.0 | Ion selective electrode Indirect potentiometry |
|  | Bicarbonate (mmol/L) | Age:  1 - 18 y  > 18 y | 20 - 31  22 - 29 | PEP carboxylase UV  (total CO_2_) |
|  | Phosphate (mg/L) | 34 - 55 | | Molybdate UV |
|  | Calcium (mg/L) | > 1 y | 85 - 105 | NM BAPTA colorimetry |
|  | 25-OH vit D (μg/L) | 30 - 60 | | Chemiluminescence immunoassays |

##

## Laboratories related to site 015

| Laboratoire Bioliance  Saint Herblain, FRANCE | Potassium (mmol/L) | Age: 8 y | 3.7 - 5.2 | Indirect potentiometry |
| --- | --- | --- | --- | --- |
|  | Bicarbonate (mmol/L) | Age: 8 y | 22 - 29 | PEP carboxylase UV |
| Laboratoire de biochimie générale  Hotel Dieu. Centre Hospitalier Universitaire (hospital laboratory).  Nantes  FRANCE | Potassium (mmol/L) | Age:  1 - 24 m  2 - 12 y  12 - 120 y | 3.7 - 5.4  3.1 - 4.7  3.4 - 4.5 | Potentiometry |
|  | Bicarbonate (mmol/L) | Age:  1 - 2 y  2 - 12 y  12 - 120 y | 20 - 27  21 - 31  24 - 34 | PEP carboxylase UV |
|  | Calcium (mmol/L) | Age:  1 - 3 y  3 - 13 y  13 - 18 y  18 - 60 y  60 - 90 y  90 - 120 y | 2.25 - 2.75  2.20 - 2.70  2.10 - 2.55  2.15 - 2.50  2.20 - 2.55  2.05 - 2.40 | Colorimetry |
|  | 25-OH vit D (ng/mL) | 30 - 60 | | Chemiluminescence immunoassays |

##

## Laboratories related to sites 202 and 301

| **Laboratory name/address** | **Parameters (units)** | **Normal ranges** | | **Analysis method** |
| --- | --- | --- | --- | --- |
| Clinical Center of Nis  (hospital Laboratory)  Niš, SERBIA | Potassium (mmol/L) | 3.1 - 5.8 | | Indirect potentiometry |
|  | Bicarbonate (mmol/L) | 18 - 25 | | Indirect method with blood gas |
|  | Phosphate (mmol/L) | 0.9 - 1.95 | | Modified phosphomolybdate (PHOS) with PMAPS and bisulfite |
|  | Calcium (mmol/L) | 2.1 - 2.75 | | Schwarzenbach's with OCPC, adapted for colorimetric determination with quinolones |
|  | 25-OH-vit D (ng/mL) | 30 - 100 | | ECLA-electro-chemiluminescence assay |
| Children’s University Hospital Bratislava (hospital laboratory).  Bratislava, SLOVAKIA | Potassium (mmol/L) | 3.5 - 5.5 | | Ion selective electrodes, indirect method (Roche) |
|  | Bicarbonate (mmol/L) | 22 - 26 | | Calculated using Hendersen-Hasselbalch equation: Derived parameter from RapidLab 1265 (Siemens) |
|  | Phosphate (µg/L) | Age:  >3m -16y  >16y F  >16y M | 20 - 200  15 - 150  30 - 400 | Photometric method with ammonium molybdate (Roche) |
|  | Calcium (mmol/L) | 2.25 - 2.85 | | Photometric method (Roche) |
|  | 25-OH-vit D (µg/mL) | 30 - 99 | | Competitive Immunoassay, Vitros Immunochemical Method, Ortho Clinical Diagnostics |

## Central laboratory

| Laboratoire de biochimie et de biologie moléculaire des Hospices Civils de Lyon, FRANCE | Bone alkaline phosphatase (µg/L) | Age  0 - 2 y  3 - 4 y  5 - 6 y  7 - 8 y  9 - 10 y  11 - 12 y  13 - 14 y  15 - 16 y  17 y  ≥18 y | F  41.9 - 107  29.5 - 108.5  21.9 - 115.4  37.1 - 147.9  42.0 - 107.6  38.6 - 111.2  13.7 - 109.8  10.2 - 72.6  5.9 - 20.0  4.9 - 26.6 | M  43.4 - 104.8  29.7 - 84.8  48.8 - 109.0  52.6 - 123.0  52.3 - 105.4  55.7 - 152.3  15.5 - 134.0  16.6 - 127.9  11.0 - 77.6  5.5 - 22.9 | Immunoluminometry  CLIA Liaison XL  (Diasorin) |
| --- | --- | --- | --- | --- | --- |
|  | Parathyroid hormone (ng/L) | 5.5 – 38.5 | | | Immunoluminometry  CLIA Liaison XL  (Diasorin) |
|  | 1,25-diOH vit D  (pmol/L) | 69 - 200 | | | Immunoluminometry  CLIA Liaison XL  (Diasorin) |

# 2. NORMAL RANGES: URINE RATIOS AND RISK OF LITHOGENESIS

| **Urine parameters (units)** | **Normal values** | | |
| --- | --- | --- | --- |
| *Urine ratios* | | | |
| Calcium/creatinine (mol/mol) [1-2] | 5 to 11 m  1 to < 2 y  2 to < 3 y  3 to < 5 y  5 to < 7 y  7 to < 10 y  10 to < 14 y  14 to 17 y  Adults | ≤ 2.2  ≤ 1.5  ≤ 1.4  ≤ 1.1  ≤ 0.8  ≤ 0.7  ≤ 0.7  ≤ 0.7  ≤ 0.5 | |
| Citrate/creatinine (mmol/mmol) [3] | M 2 to <7 y  M 7 to <13 y  M 13 to <18 y  M Adults  F 2 to <7 y  F 7 to <13 y  F 13 to <18 y  F Adults | ≥ 0.142  ≥ 0.082  ≥ 0.052  ≥ 0.052*  ≥ 0.171  ≥ 0.154  ≥ 0.127  ≥ 0.127* | |
| *Risk of lithogenesis* | | | |
| Calcium/citrate (mmol/mmol) [4] | All | | ≤ 3 |

* The reference limit for adolescents also applied to adults according to expert opinion

1. Matos V, van Melle G, Boulat O et al (1997) Urinary phosphate/creatinine, calcium/creatinine, and magnesium/creatinine ratios in a healthy pediatric population. J Pediatr 131:252-257.

2. Frey J, Daudon M, Raby N et al (2001) Valeur sémiologique des paramètres biochimiques urinaires. Ann Biol Clin 59:13-25.

3. Kirejczyk JK, Porowski T, Konstantynowicz J et al (2014) Urinary citrate excretion in healthy children depends on age and gender. Pediatr Nephrol 29:1575-1582.

4. Parent X BG, Brignon P (1999) Lithiase oxalocalcique. Relation entre facteurs de risque biochimiques et phase cristalline du calcul. Prog Urol 6:1051-1056.
