# Supplementary material for: Safety, efficacy, and acceptability of ADV7103 during 24 months of treatment: an open-label study in pediatric and adult patients with distal renal tubular acidosis
Source: Pediatr Nephrol. 2021 Feb 26;36(7):1765–74. doi: 10.1007/s00467-020-04873-0 (PMC8172410; doi:10.1007/s00467-020-04873-0)
Supplement: Supplementary file 2 — (PPTX 57.5 kb). [file 467_2020_4873_MOESM2_ESM.pptx]

## Slide 1
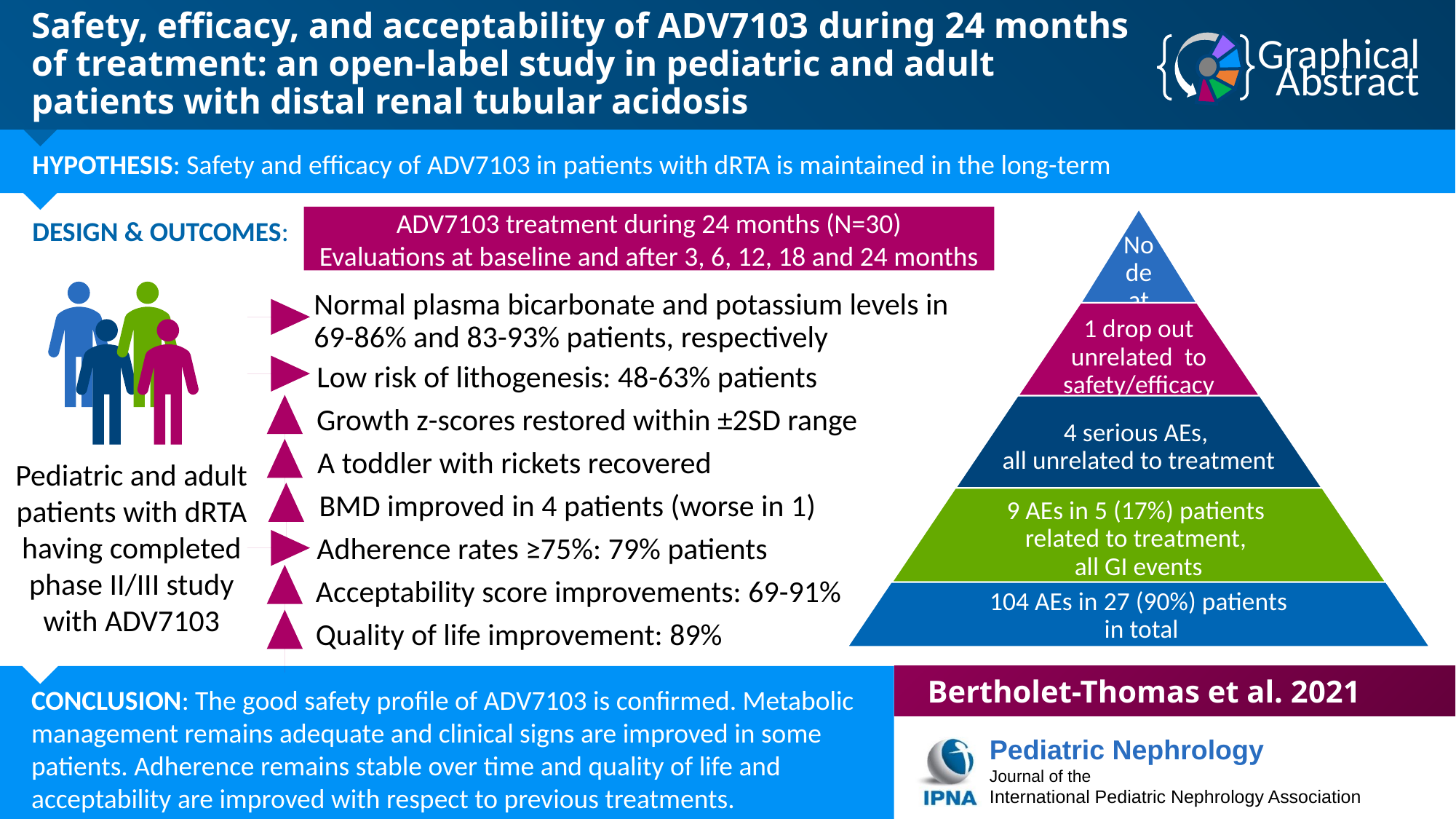

Safety, efficacy, and acceptability of ADV7103 during 24 months of treatment: an open-label study in pediatric and adult patients with distal renal tubular acidosis
HYPOTHESIS: Safety and efficacy of ADV7103 in patients with dRTA is maintained in the long-term
ADV7103 treatment during 24 months (N=30)
Evaluations at baseline and after 3, 6, 12, 18 and 24 months
DESIGN & OUTCOMES:
Normal plasma bicarbonate and potassium levels in
69-86% and 83-93% patients, respectively
Low risk of lithogenesis: 48-63% patients
Growth z-scores restored within ±2SD range
A toddler with rickets recovered
Pediatric and adult patients with dRTA
having completed phase II/III study with ADV7103
BMD improved in 4 patients (worse in 1)
Adherence rates ≥75%: 79% patients
Acceptability score improvements: 69-91%
Quality of life improvement: 89%
Bertholet-Thomas et al. 2021
CONCLUSION: The good safety profile of ADV7103 is confirmed. Metabolic management remains adequate and clinical signs are improved in some patients. Adherence remains stable over time and quality of life and acceptability are improved with respect to previous treatments.
